# Precision needle-punch tumor enrichment from paraffin blocks improves the detection of clinically actionable genomic alterations and biomarkers

**DOI:** 10.3389/fonc.2024.1328512

**Published:** 2024-02-20

**Authors:** Douglas I. Lin, Richard S. P. Huang, Ioannis Ladas, Rachel B. Keller, Nimesh R. Patel, Sotirios Lakis, Brennan Decker, Tyler Janovitz, Douglas A. Mata, Jeffrey S. Ross, Jo-Anne Vergilio, Julia A. Elvin, Roy S. Herbst, Philip C. Mack, Jonathan K. Killian

**Affiliations:** ^1^ Department of Pathology and Diagnostic Medicine, Foundation Medicine, Inc., Cambridge, MA, United States; ^2^ Foundation Medicine GmbH, Pathology Department, Penzberg, Germany; ^3^ Department of Medical Oncology, Yale School of Medicine, Yale Cancer Center, New Haven, CT, United States; ^4^ Division of Hematology and Oncology, Department of Medicine, Tisch Cancer Institute at Mount Sinai, New York, NY, United States

**Keywords:** tumor enrichment, tumor purity, biomarker, molecular diagnosis, FoundationOne^®^CDx, tumor microdissection, next generation sequencing, biomarkers

## Abstract

**Background:**

While many molecular assays can detect mutations at low tumor purity and variant allele frequencies, complex biomarkers such as tumor mutational burden (TMB), microsatellite instability (MSI), and genomic loss of heterozygosity (gLOH) require higher tumor purity for accurate measurement. Scalable, quality-controlled, tissue-conserving methods to increase tumor nuclei percentage (TN%) from tumor specimens are needed for complex biomarkers and hence necessary to maximize patient matching to approved therapies or clinical trial enrollment. We evaluated the clinical utility and performance of precision needle-punch enrichment (NPE) compared with traditional razor blade macroenrichment of tumor specimens on molecular testing success.

**Methods:**

Pathologist-directed NPE was performed manually on formalin-fixed, paraffin embedded (FFPE) blocks. Quality control of target capture region and quantity of residual tumor in each tissue block was determined via a post-enrichment histologic slide recut. Resultant tumor purity and biomarker status were determined by the computational analysis pipeline component of the FDA-approved next-generation sequencing (NGS) assay, FoundationOne^®^CDx. Following NPE implementation for real-world clinical samples, assay performance and biomarker (MSI, TMB, gLOH) detection were analyzed.

**Results:**

In real-world clinical samples, enrichment rate via NPE was increased to ~50% over a 2.5-year period, exceeding the prior use of razor blade macro-enrichment (<30% of cases) prior to NPE implementation due to proven efficacy in generating high quality molecular results from marginal samples and the ease of use for both pathologist and histotechnologists. NPE was associated with lower test failures, higher computational tumor purity, and higher rates of successful TMB, MSI and gLOH determination when stratified by pre-enriched (incipient) tumor nuclei percentage. In addition, challenging cases in which tumor content was initially insufficient for testing were salvaged for analysis of biomarker status, gene amplification/deletion, and confident mutant or wild-type gene status determination.

**Conclusions:**

Pathologist-directed precision enrichment from tissue blocks (aka NPE) increases tumor purity, and consequently, yields a greater number of successful tests and complex biomarker determinations. Moreover, this process is rapid, safe, inexpensive, scalable, and conserves patient surgical pathology material. NPE may constitute best practice with respect to enriching tumor cells from low-purity specimens for biomarker detection in molecular laboratories.

## Introduction

Cancer samples with low tumor purity are often inadequate for molecular testing. Complex biomarkers, such as genomic loss of heterozygosity (gLOH) score, a measure of homologous recombination deficiency (HRD)​, tumor mutational burden (TMB)​, and microsatellite instability (MSI),​ require a higher tumor purity for confident detection, with limit of detection (95% hit rate) ranging from 9.8% tumor purity for MSI-high across solid tumors to 35% tumor purity for gLOH in ovarian cancer via FoundationOne^®^CDx (F1CDx).​ High tumor purity is also needed to detect gene copy-number changes (amplification or homozygous deletion) as well as to confidently determine wild-type or mutant status of actionable genes. Traditionally, while single nucleotide variants can typically be identified from samples with relatively low tumor content, many next-generation sequencing (NGS)-based assays require a minimal tumor nuclei percentage (TN%) for testing, such as 20% (1), and methods to enrich the tumor content (e.g., macro- or micro-dissection) prior to testing may be attempted if a pre-defined minimum TN% is not met. However, enrichment methods may vary, and more generally, practical, and effective methods to increase TN% from pathology specimens are needed to achieve biomarker results and to maximize patient matching to approved therapies and/or clinical trial enrollment.

Clinical solid tumor specimens have areas with both malignant and benign cells, often in close proximity to one another and to varying degrees intermixed. For this reason, any surgical biopsy or resection specimen is at potential risk of harboring a substantial non-neoplastic nucleated cell component, which can mask the detection of informative somatic genomic alterations in tumor cells. Therefore, prior to genomic testing, the standard practice is to determine a pre-test incipient tumor nuclear percentage (TN%) by a pathologist review of histology, and if TN% is low, the sample may be rejected (Tissue Not Sufficient for Analysis [TIFA]), or it may run at risk of a generating computationally low tumor purity (LTP) result. More generally, tumor enrichment may be electively directed by the pathologist to obtain molecular results from a specific tumor spatial target. Both TIFA and low tumor purity (LTP) results (i.e., when sequencing is performed on a low TN% specimen that appears computationally low tumor purity, indicating an increased risk of false negative results) may cause test delays, failure of testing, need for repeat analysis, patient re-biopsy and increased testing turnaround time (TAT) without generating meaningful molecular results. Ineffective enrichment methods increase the risk for repeated NGS test or re-biopsy, increase sequencing costs, consume additional laboratory as well as clinical resources, and thereby, increase the dissatisfaction of patients, oncologists, or referring pathology laboratories.

After a tumor is sequenced, a calculated tumor purity may also be determined by computational methods independently of a prior pathologist’s assessment. Similar to TN%, a minimum computational tumor purity threshold (i.e., 20% or 30%) may be required to confidently report complex biomarkers such as gLOH, MSI, TMB, copy number gains and losses, certain fusions constructs and tumor-specific RNA expression patterns. Specimens with low computational tumor purity may result in biomarker test failure or in false-negative results. Therefore, precise, safe, efficient, and scalable methods are needed to salvage cases with low tumor purity and to confidently determine biomarker status.

Current methods of tumor enrichment before sequencing and biomarker detection include semi-automated systems such as laser capture microdissection and Millisect, or manual approaches such as macrodissection with a razor blade or scalpel and needle core enrichment (2). Out of these, manual macrodissection enrichment with a scalpel or razor blade by a histotechnologist is a common method historically used in many molecular pathology laboratories. Macrodissection-based enrichment is historically and originally designed for unstained glass slide recuts, and it can be extended to paraffin blocks. It uses a blade to select tumor tissue away from benign elements by manual dissection of the tumor on an unstained slide or block. Manual razor blade-guided macrodissection has inherent challenges in capturing small (<2 mm) targets embedded in surrounding non-tumor tissue and is labor intensive. Post-enrichment histologic quality control view of target capture following manual macrodissection from tissue blocks cannot be reliably performed to verify accuracy of enrichment, and untargeted tissue on slide or block may go to waste.

In contrast to macroenrichment via razor blade, enrichment may also be achieved via needle-punch enrichment (NPE) of tumor area of interest in FFPE blocks (3, 4). In our current practice, we employ a single-use, blunt-tip, thin-walled, stainless steel needle for enrichment to precisely capture microscopic tumor targets (<1mm) isolated from surrounding non-tumor tissue in FFPE blocks. In addition, this enables post-enrichment quality control analysis of post-punch H&E images of target capture to determine accuracy of enrichment and eliminates patient specimen waste by maximizing tissue and tumor preservation. Technically, it is fast, facile, and scalable. We previously described the process qualification and validation of NPE from paraffin blocks for comprehensive genomic profiling (CGP) (5). Here, we describe the institution of NPE to salvage cases with low tumor purity for genomic testing, and we retrospectively analyze the clinical utility and molecular assay performance after NPE implementation compared with razor blade macroenrichment in clinical workflow samples that were submitted for CGP via F1CDx.

## Methods

### Comprehensive genomic profiling

F1CDx is an FDA-approved, NGS-based CGP, *in vitro* diagnostic device based on hybridization-based capture technology and NGS for the detection of substitutions, short insertion and deletion alterations, copy-number alterations including amplifications and homozygous deletions, and select rearrangements in 324 genes, and of genomic signatures, including MSI, TMB, and gLOH/HRD for tubo-ovarian and peritoneal carcinomas. F1CDx methods have been described previously (6). Sheared genomic DNA isolated from FFPE tumor tissue specimens was used for library construction, hybrid capture, and targeted sequencing. Sequence data were analyzed using proprietary software developed by Foundation Medicine, and variant calling was performed as previously described (7). Computational purity was assessed after sequencing based on the degree of aneuploidy.

### Precision needle punch enrichment

**Figure 1:**
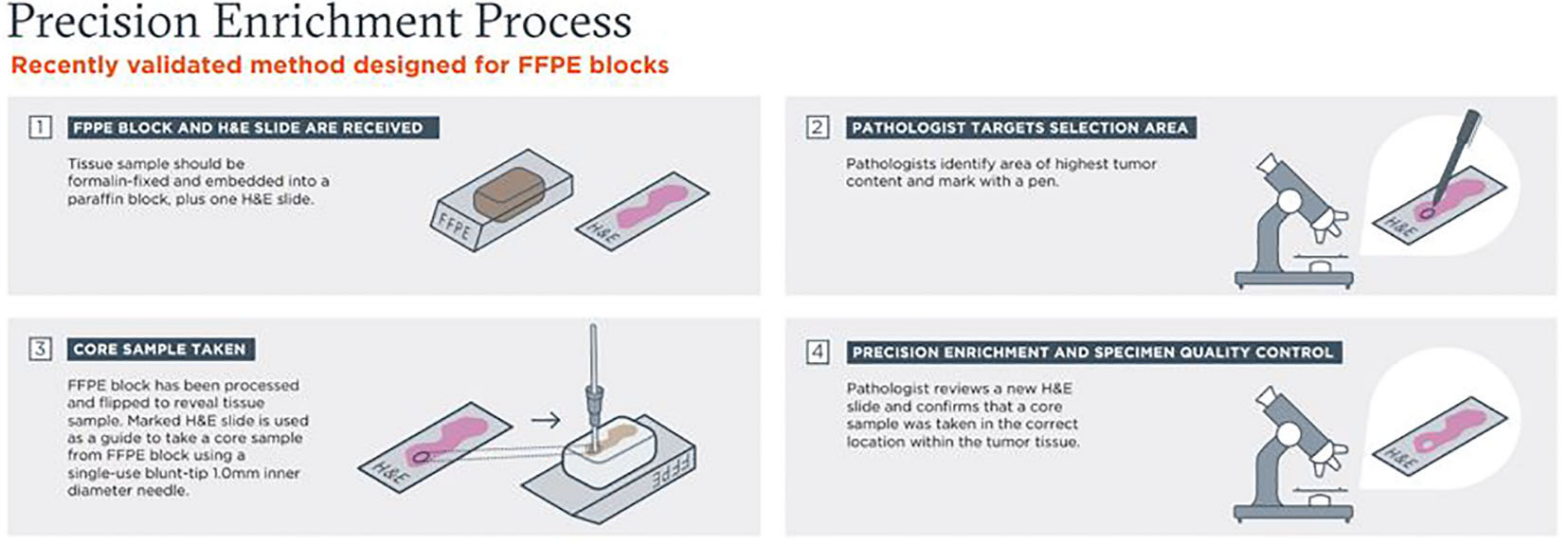
Overview of pathologist-directed, precision needle punch enrichment (NPE), a quality-controlled process.

An overview of the pathologist-directed NPE, quality-controlled process, is highlighted in **Figure 1**. NPE was performed on FFPE tissue blocks with a corresponding matching H&E slide. Pathologists at Foundation Medicine identified a spatial target with desired tumor content and marked it with a pen. Up to 5 areas could be marked and punched per tissue block. The marked H&E slide was then used as a guide to take needle core punch samples from FFPE blocks using a single-use blunt-tip, thin-walled 14 gauge (~1.4 mm) inner diameter needle by histotechnologists. After taking core samples, a quality control post-punch H&E slide was prepared, and a pathologist confirmed that core sample(s) were taken in the correct location within the tumor tissue. All types of FFPE tissue blocks (excisions, small biopsies, FNA/EBUS cell blocks) were previously validated and were eligible for NPE as long as the sample was predicted to have a 0.6 mm^3^ enriched tissue volume. This volume requirement was typically achieved with 1 punch, but additional punch(es) for small samples could be added at the pathologist’s discretion. Following NPE, methodology is the same as for razor blade (RBE) and unenriched (UnE) samples. The native FFPE UnE or enriched tissue (both NPE and RBE) is placed into a digest tube, at which point all samples are processed the same through digestion/deparaffinization, DNA extraction, and downstream steps.

### NPE method validation and qualification

A process qualification/validation study was previously performed and described to demonstrate non-inferiority and compatibility of NPE specimen processing with the extant DNA extraction chemistry and methodology of F1CDx (5). Computational purities were compared between no enrichment (UnE, n=46), straight razor blade macro-enrichment (RBE, n=30) or NPE (n=47). Computational purity was determined by the computational analysis pipeline component of F1CDx. Post-enrichment H&E QC slides confirmed spatial target capture for the NPE arm. Given the different specimen dimensions of punches versus macroenrichment curls, we also evaluated how punches might affect the kinetics of sample digestion using the existing standard extraction conditions.

### NPE real-world assay metrics from clinical workflow specimens

Following NPE process qualification and training of pathologists and histotechnologists, we implemented NPE for F1CDx on real-world clinical samples in our Cambridge, MA, Foundation Medicine molecular laboratory in the second quarter (Q2) of 2020. When analyzing clinical samples, pathologists could choose NPE, RBE, or no enrichment at their professional discretion. F1CDx assay metrics, including TIFA, computational purity, and biomarker status, were compared in clinical workflow samples between unenriched, RBE and NPE samples over a 2.5-year period. F1CDx assay metrics were compared for RBE samples prior to NPE implementation with NPE samples.

### Biomarker analysis

MSI, TMB, and gLOH statuses were determined by the F1CDx computational pipeline. To determine MSI status, F1CDx employs a fraction-based (FB) MSI algorithm to categorize a tumor as MSI-H, which calculates the fraction of unstable microsatellite loci based on an analysis across >2,000 loci. TMB was determined on 0.79–1.14 Mb of sequenced DNA (8). We used validated methods for detecting gLOH, a surrogate marker for HRD and PARPi effectiveness, in ovarian cancer (9, 10). Patients’ solid tumors receive biomarker statuses as “pass” with corresponding value, such as MSI-H and MSS, TMB and gLOH scores, or alternatively “Fail/Cannot Be Determined” due to a quality control (QC) failure, such as due to low tumor purity.

## Results

### NPE implementation on clinical samples and assay test failures

**Figure 2:**
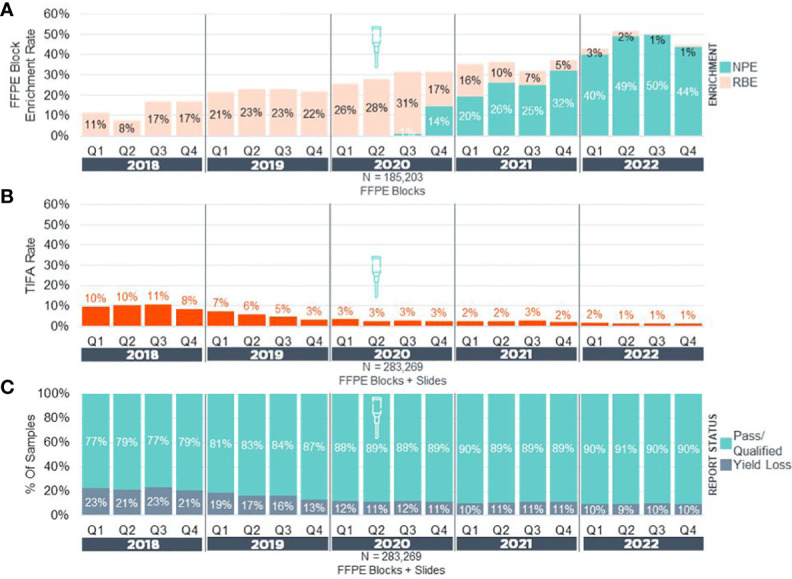
Precision Enrichment Real-World Experience Pan-F1CDx. **(A)** Overall enrichment rates in real-world samples (NPE + RBE) for FFPE blocks received from 2018-2022 (N = 185,203). **(B)** Tissue Insufficient For Analysis (TIFA) rates in real-world samples (FFPE Blocks + Slides) before and after implementation of NPE. **(C)** Reporting status rates in real-world samples (FFPE Blocks + Slides) before and after implementation of NPE. Yield Loss = Unsuccessful Samples + TIFA. Approximate date of NPE implementation is denoted by a needle icon. NPE, Needle Punch Enrichment; RBE, Razor-Blade Macro-Enrichment.

After making NPE available for use (**Figure 1**) on real-world clinical cases, pathologists were given the choice of no enrichment, NPE, or razor blade macro-enrichment (RBE) for a 30-month period. Subsequently, we retrospectively assessed block enrichment rates and metrics of F1CDx assay success, such as: 1) tissue insufficient for analysis (TIFA) before sequencing, and 2) rates of successful (pass and qualified) and unsuccessful reports (**Figure 2**) and compared them to pre-NPE implementation data. Prior to NPE implementation, block enrichment rate via RBE had been increasing from ~10% to ~30% of cases over 30-month period in an effort to optimize tissue handling and to increase the proportion of successful reports and biomarker determination. During the same period, TIFA rates decreased from 10% to 3%, while overall proportion of pass/qualified reports increased from 77% to 89%. After NPE implementation, enrichment rate dramatically increased to as high as 50% of cases, the vast majority (>95%) of which were enriched via NPE (**Figure 2A**). During the same 2.5-year period, overall TIFA rates further decreased by 66.7% (from 3% down to 1%) in our Cambridge laboratory (**Figure 2B**), while overall rates of unsuccessful reports (yield loss) across all laboratory sites further decreased by up to 18% (from 11% to down to 9-10%) with overall increase in proportion of pass/qualified reports to 90-91% (**Figure 2C**).

### Non-inferiority computational purity in clinical workflow samples following NPE implementation

**Figure 3:**
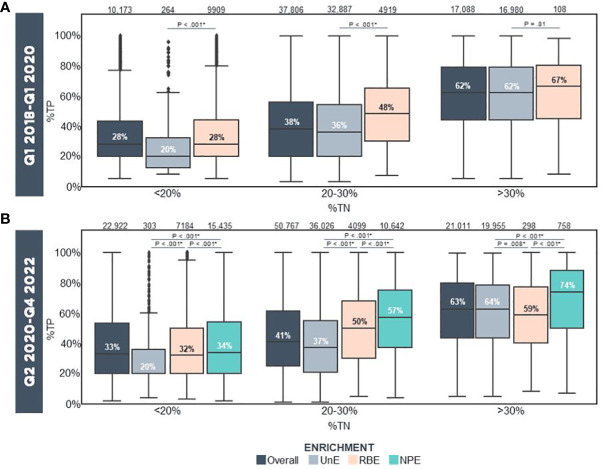
Computational Purity In Real-World Samples Prior To/Following Clinical Implementation Of Precision Enrichment **(A)** Computational purity of all reportable (i.e., QC Pass/Qualified), UnE, and RBE samples stratified by incipient TN% prior to NPE implementation (Q1 2018-Q1 2020; N = 65,067 FFPE Blocks). **(B)** Computational purity of all reportable, UnE, RBE and NPE samples stratified by incipient TN% following NPE implementation (Q2 2020-Q4 2022; N = 94,700 FFPE Blocks). Median %TP is indicated. The number of samples in each category is indicated above the bars. Statistical analysis was performed using the T Test with the Benjamini-Hochberg Procedure for p-value multiple hypothesis corrections (*p <.05). NPE, Needle Punch Precision Enrichment; RBE, Razor-Blade Macro-Enrichment; %TN, Tumor Nuclei Percentage; %TP, Computational Tumor Purity; UnE, Unenriched.

We further assessed non-inferiority biomarker experience from tissue blocks following NPE implementation compared to UnE and RBE samples over a 2-year period. The following metrics and biomarkers were analyzed and stratified by incipient (pre-enriched) tumor nuclei percentage (%TN): 1) Computational purity as assessed by the F1CDx pipeline, 2) MSI, 3) TMB, and 4) gLOH biomarkers. For these analyses, incipient pre-enriched TN% were stratified as <20%, 20-30%, and >30%. Samples with incipient <20% TN are at a very high risk for assay failure; those between 20-30% TN may yield a low tumor purity result, whereas those with >30% may be anticipated to yield passing computational tumor purity in most cases. Prior to NPE implementation, median computational tumor purity for overall sequenced samples were 28%, 38% and 62% for tumors with incipient TN% of <20%, 20-30% and >30%, respectively (**Figure 3A**). Within the same timeframe, median computational purity of unenriched samples was 20%, 36% and 62%, while computational tumor purity of macro RBE samples was 28%, 48% and 67% as stratified with the same incipient TN% (**Figure 3A**). In contrast, following NPE implementation, median computational purity for overall sequenced samples was 33%, 41% and 63% for tumors with incipient TN% of <20%, 20-30% and >30% (**Figure 3B**). Direct comparison of median computational tumor purity of unenriched and NPE samples revealed higher computational purity in NPE samples at 34%, 57% and 74% for NPE samples with <20%, 20-30% and >30% incipient TN%, respectively, compared with 20%, 37% and 64% stratified with <20%, 20-30% and >30% incipient TN% for UnE samples (**Figure 3B**). Median computational purity was significantly higher in NPE samples compared to UnE (Post-NPE) and RBE across all incipient TN% categories [*p <.001 UnE (Post-NPE) –versus– NPE. ^§^p <.001 RBE –versus– NPE].

### Non-inferiority biomarker status in clinical samples following NPE

**Figure 4:**
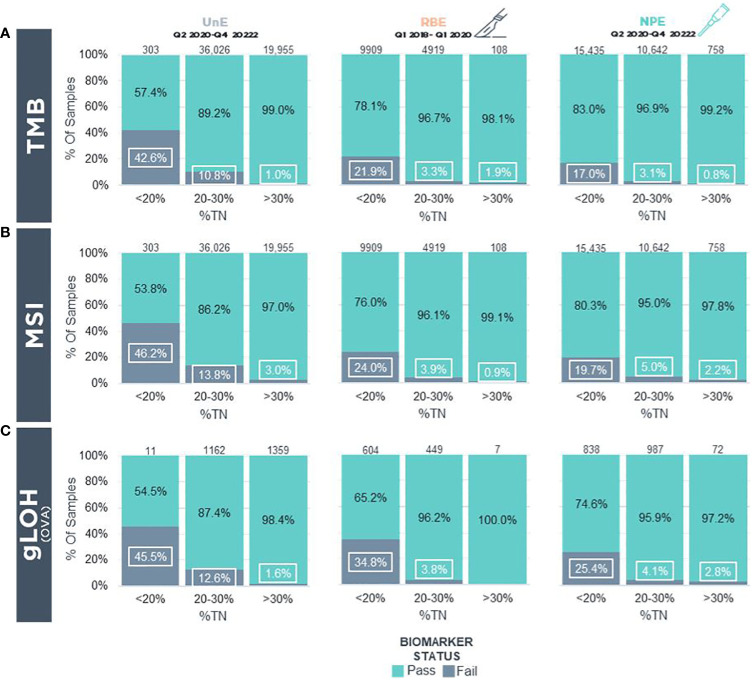
Biomarker Status Determination In Real-World FFPE Tumor Blocks Following Clinical Implementation Of Precision Enrichment **(A)** TMB and **(B)** MSI biomarker reporting status in real-world samples for reportable (i.e., QC Pass/Qualified) UnE (Left), RBE (Middle), and NPE (Right) FFPE blocks stratified by incipient TN%. **(C)** gLOH biomarker reporting status in real-world samples in reportable UnE (Left), RBE (Middle), and NPE (Right) ovarian, tubo-ovarian, and peritoneal carcinoma FFPE blocks stratified by incipient TN%. LOH, Loss of Heterozygosity; MSI, Microsatellite Instability; NPE, Needle Punch Enrichment; OVA, Ovarian/Tubo-ovarian/Peritoneal Carcinoma; RBE, Razor-Blade Macro-Enrichment; TMB, Tumor Mutational Burden; TN%, Tumor Nuclei Percentage; UnE, Unenriched.

We next determined the success rate of biomarker (TMB, MSI, gLOH) status determination in UnE, NPE, and RBE samples. In UnE samples, TMB could not be determined in 42.6%, 10.8%, and 1.0% of cases with incipient TN% of <20%, 20-30% and >30%, respectively (**Figure 4A**). In contrast, NPE had a reduction in “TMB cannot be determined status” to 17.0%, 3.1%, and 0.8% across the respective incipient (pre-enriched) %TN categories (**Figure 4A**). As a comparison, in a 2-year period prior to NPE implementation, “TMB cannot be determined” status frequency with RBE macroenrichment was 21.9%, 3.3%, and 1.9% of cases, with incipient TN% of <20%, 20-30% and >30%, respectively (**Figure 4A**).

Similar to TMB, there was a reduction in “MSI cannot be determined” status with NPE across different incipient TN% compared with UnE samples. In UnE samples, MSI status could not be determined in 46.2%, 13.8%, and 3.0% with incipient TN% of <20%, 20-30%, and >30%, respectively (**Figure 4B**). In contrast, NPE reduced “MSI cannot be determined status” to 19.7%, 5.0%, and 2.2% across the respective incipient %TN categories (**Figure 4B**). In contrast, prior to NPE implementation, MSI cannot be determined via RBE macroenrichment in 24.0%, 3.9%, and 0.9% of cases, with incipient TN% of <20%, 20-30%, and >30%, respectively (**Figure 4B**).

Finally, we assessed success rates of the gLOH biomarker, which is a complex biomarker for HRD in ovarian cancer that has a limit of detection of approximately 30% computational tumor purity. Metrics for gLOH status determination was restricted to tubo-ovarian and peritoneal carcinomas. In UnE samples, the gLOH biomarker could not be determined in 45.5%, 12.6%, and 1.6% of cases with incipient TN% of <20%, 20-30% and >30%, respectively (**Figure 4C**). In contrast, NPE reduced “gLOH cannot be determined” status to 25.4%, 4.1%, and 2.8% with incipient TN% of <20%, 20-30%, and >30% (**Figure 4C**). By comparison, the gLOH biomarker cannot be determined with RBE in 34.8%, 3.8%, and 0% of cases, with incipient TN% of <20%, 20-30%, and >30%, respectively (**Figure 4C**). The overall biomarker and computational purity results following NPE suggest that NPE may enhance biomarker status determination, especially in the incipient, pre-enriched low tumor purity category (i.e., <20% TN), in which biomarkers would otherwise not be able to be confidently determined.

### Salvaging challenging clinical workflow cases

**Figure 5:**
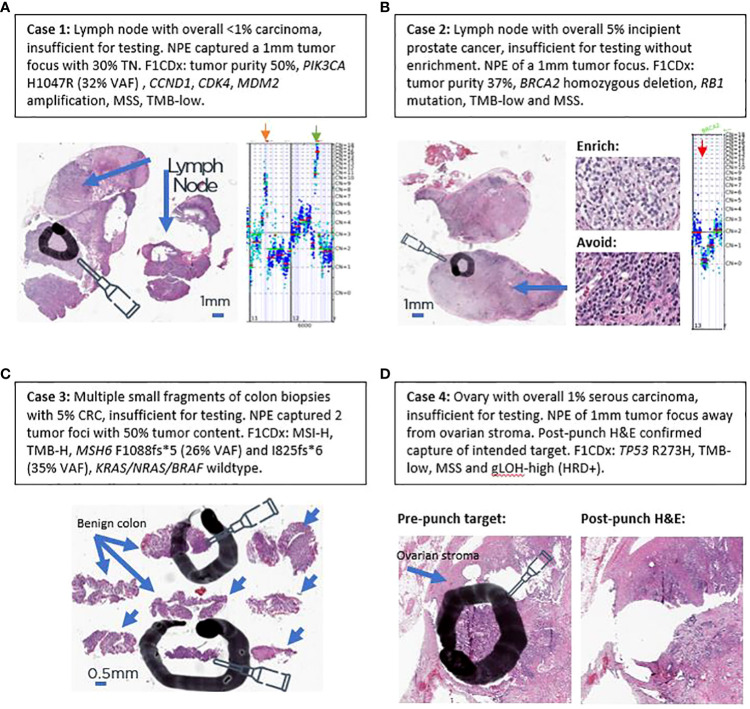
Examples of salvaging four challenging real-world clinical cases. **(A)** Case 1. **(B)** Case 2. **(C)** Case 3. **(D)** Case 4. Black circles and ovals denote intended tumor target areas by NPE (needle). Blue arrows denote areas of benign tissue to avoid. Orange arrow denotes *CCND1* amplification on copy number plot of case 1. Green arrow denotes *CDK4* and *MDM2* amplification on copy number plot of case 1. Red arrow denotes biallelic *BRCA2* loss in copy number plot of case 2.

In addition to the aggregate increase in percentage of cases with successful testing, the impact at the patient level of this technique can be better appreciated by examining real-world clinical examples of challenging cases in which tumor content was initially insufficient for testing but were salvaged by NPE. Case #1 was from a lymph node of a 59-year-old woman with history of both lung and breast carcinomas. Mediastinal lymph node biopsy demonstrated a minute 1-mm focus of metastatic carcinoma (**Figure 5A**), which represented <1% of the total nucleated cells. Without enrichment, the sample would not have met the testing requirement of ≥20% tumor content, and the tumor focus was too small to be rescued by standard RBE. NPE captured 30% tumor cells for F1CDx testing, which revealed a computational tumor purity of 50%. An activating *PIK3CA* H1047R mutation (32% VAF) was also identified, which yielded an alpelisib FDA-approved CDx therapy association based on the SOLAR-1 trial (11). TMB and MSI biomarker status were successfully determined as TMB-low and microsatellite stable, and amplifications in *CCND1*, *CDK4*, *MDM2* and several other genes were also identified.

Case #2 is from 53-year-old man with history of prostatic and renal cancers. A subaortic (station 5) lymph node with metastatic prostate cancer was submitted for sequencing, which harbored 5% tumor cells (**Figure 5B**). NPE confidently captured a 1-mm high-purity tumor target (**Figure 5B**). F1CDx revealed a computational purity of 37% and homozygous *BRCA2* loss (**Figure 5B**), which has been described in 1–2% of primary and 2–3% of metastatic prostate cancers (12–14). Based on the Phase 3 PROfound study (15), olaparib was reported as a CDx+ therapy association. In addition to *BRCA2* loss, the tumor exhibited low TMB, was microsatellite stable, and harbored a co-occurring *RB1* mutation. Since sensitive detection of copy-number changes requires adequate tumor purity, this case illustrates the utility of NPE in increasing the tumor purity of a specimen for sensitive detection of a tumor suppressor gene loss (*BRCA2*).

Case #3 is from a 76-year-old patient with multiple small colon biopsies, that had been collected in one tissue block. Only 2 of 8 biopsy fragments contained invasive carcinoma (**Figure 5C**), which comprised 5% of total nucleated cells. NPE captured two needle punches of 50% tumor content. F1CDx revealed MSI-H and TMB-high biomarker statuses and wild-type *KRAS, NRAS*, and *BRAF*. Two distinct mutations in *MSH6* were identified: 1) *MSH6* F1088fs*5 (3261_3262insC, 25.95% VAF) and *MSH6* I825fs*6 (34.96% VAF), raising the possibility of Lynch Syndrome (16). Absence of *BRAF* V600E indicated that *MLH1* promoter hypermethylation was not the likely mechanism of MSI-H (17). Based on MSI-H and TMB-H biomarker statuses, FDA-approved pembrolizumab was listed as CDx-associated therapy on the patient’s report (18, 19). Wild-type *KRAS/NRAS* statuses were also CDx-associated findings for FDA-approved cetuximab and panitumumab. Case #3 highlights the utility of NPE in small biopsies with low pre-enriched, incipient tumor purity for the detection of MSI and TMB biomarker as well as of wild-type *KRAS*/*NRAS*/*BRAF* statuses in small gastrointestinal biopsies.

Case #4 is of a 69-year-old woman with advanced tubo-ovarian high-grade serous carcinoma. One of the ovaries was submitted for testing and harbored 1% tumor cells due to associated dense ovarian stroma and inflammation (**Figure 5D**). As assessed by a pathologist, RBE could not attain 30% tumor purity required for the gLOH biomarker, which is a surrogate marker for HRD. NPE was performed for F1CDX testing (**Figure 5D**), which revealed a computational purity of 40%. No alterations in *BRCA1/2* were identified; however, this tumor had a *TP53* R273H hotspot mutation and a high gLOH score, which has been shown to be associated with sensitivity to the PARP inhibitor rucaparib in platinum-sensitive, *BRCA*1/2 wild-type ovarian cancer (9, 10). Case #4 illustrates the utility of NPE for the detection of a complex biomarker that requires at least 30% tumor purity for reporting.

## Discussion

It is standard practice for pathologists to perform microscopic assessment of the tumor cell content prior to genomic testing to determine whether the sample is sufficient for testing (20). If tumor content is deemed to be insufficient, enrichment may be pathologist-directed to increase the likelihood of a successful CGP test. Here, we present data that NPE is superior to RBE, a legacy method used in most molecular pathology laboratories, and NPE may constitute a new gold standard enrichment method for genomic testing. We demonstrate the value of NPE in increasing the proportion of specimens that are sufficient for NGS-based molecular profiling with F1CDx in excess of what was previously achieved with RBE, and for improving the reportability of complex biomarkers (MSI, TMB, gLOH/HRD) especially for samples that were submitted close to the minimal tumor content as well as in samples estimated to have incipient TN% >30%. In specimens where incipient TN% may appear >30%, NPE ensures that a desired target is tested and reduces inclusion of inhibitory or undesired spatial regions – both recognized and potentially unrecognized during pathology review. These data support that NPE has clinical utility in enhancing genomic alteration and biomarker detection in tumor samples from patients that may benefit from FDA-approved therapies with requisite biomarker identification and/or companion diagnostics.

Advantages of NPE over traditional manual microdissection techniques are that it is scalable, rapid, practical, designed for real-world clinical FFPE tissue blocks and can be routinely implemented in standard diagnostic laboratories. Quality control monitoring in this setting can be readily applied through use of a post-punch H&E to ensure accuracy and that the intended tumor area for enrichment was indeed targeted via histologic visual confirmation. In our experience reviewing post-punch quality control H&Es, NPE is highly accurate with an off-target rate of NPE and percentage necessary to re-punch after analysis of control slides of <1%. Other advantages of NPE compared to RBE include maximal tissue conservation, sparing the block from undergoing thick curls and potential block exhaustion, and minimizing risk to laboratory technicians, who forgo use of additional microtomy and sharp razor blades. Compared to semi-automated systems such as laser capture microdissection and Millisect, NPE does not require costly equipment and reagents, is less time consuming, and is scalable in a high-volume laboratory. For these reasons, NPE is an efficient, cost-effective alternative that does not require microtome usage.

Despite advances over the years in NGS-based CGP techniques and analytics that have significantly increased both assay sensitivity and specificity for routine variants such as single nucleotide substitutions, there is still a substantial need for tumor enrichment in modern practice. In addition to the more complex assays assessed in this report (MSI, TMB and gLOH/HRD), a variety of other diagnostic strategies of increasing interest in oncology can benefit from a rapid, easy to implement tumor enrichment approach. Potential candidates would include quantitative assays that require copy number measurements over background levels, such as mRNA or microRNA expression profiling or DNA copy number abnormalities, where the absence of overt sequence differences (i.e. mutations) requires substantially higher tumor content to detect differences. Increased interest in tumor microenvironment cellular compositions, whether assessed at the protein, DNA or RNA level, may be advanced both by increased tumor cell enrichment (to examine increased infiltration in tumor-rich areas while excluding neighboring non-tumor features) as well as dedicated punches of tumor-adjacent cell populations that may have relevance to immune oncology therapeutics. Other potential applications include analysis of tumor spatial heterogeneity. Currently, the ability to implement such new diagnostic approaches is limited by the increased costs and turn-around time associated with more exacting dissection practices and pathologist oversight. A simple, but rapid and effective enrichment approach, such as pathologist-directed NPE, may ultimately accelerate adoption rates of complex but high-utility tests.

At Foundation Medicine, we had been working to optimize our tissue handling and to educate external pathology laboratories on how to process specimens for molecular testing for 7+ years prior to NPE implementation. In addition, the launch and reporting of complex biomarkers, such as TMB, MSI and gLOH/HRD may have led to an increased adoption of macroenrichment via RBE by Foundation Medicine pathologists as those biomarkers require a higher tumor content for detection. Consequently, all these measures may have resulted in increased proportion of successful reports and decrease of TIFA rates prior to the adoption of NPE. Following implementation, NPE yielded an additional gain on top of all the other measures previously taken to increase assay and biomarker success and to reduce tissue insufficiency for molecular testing. Furthermore, the willingness of pathologists to adopt NPE very liberally may be a combination of its proven success and the lack of a downside from a processing perspective.

NPE has now changed our molecular pathology practice for CGP via F1CDx, a high-volume FDA-approved CGP assay with multiple CDx indications in the treatment of solid tumors, as evidenced by an increase in our enrichment rates from ~30% prior to NPE implementation to ~50%, 30-months following implementation. The data presented here is from clinical samples that were processed in our headquarters laboratory in Cambridge, MA, the first laboratory site where we validated and implemented NPE. We have previously presented data that NPE may also be useful in prospective clinical trial samples (5). Consequently, after implementation on clinical samples, we have also started performing NPE on clinical trials samples, and we have further validated and implemented NPE at other satellite laboratories in the Research Triangle Park (RTP), NC, USA and in Penzberg, Germany. Data from our RTP laboratory shows similar increase in enrichment rates from 21% prior to NPE implementation up to 46% following 1 year of NPE implementation, with a concurrent decrease in samples with tissue/tumor insufficient for testing (TIFA) from 8.3% to 4.7%, respectively. Hence, implementation of NPE can be considered in any clinical laboratory setting with adequate validation, and it may result in immediate clinical utility.

In our current practice, RBE can still be performed, but our preferred enrichment method for tissue blocks is precision enrichment (aka NPE), which is the current method of enrichment in >90% of tissue blocks, when enrichment is performed. All sample types (excisions, small biopsies, FNA/EBUS cell blocks) were previously validated and are eligible for NPE at Foundation Medicine as long as the enriched sample is predicted to have a 0.6 mm^3^ tissue volume. One punch is typically sufficient to achieve enough DNA yield for CGP analysis, but additional punch(es) for small samples may be added at pathologist’s discretion. In this procedure, ~1-mm diameter core(s) are precisely placed in the block, which can salvage samples that would otherwise be insufficient for testing, as highlighted by our 4 examples in **Figure 5**. RBE may still be performed at the discretion of the Foundation Medicine reviewing pathologist, but we currently tend to do RBE only when unstained slides are provided rather than a FFPE tumor block (required for NPE) or when the predicted enriched tissue volume is less than 0.6 mm^3^ by NPE. Whether to enrich a sample or not is also at the discretion of the reviewing pathologist after giving careful consideration of other competing factors, such as initial (unenriched) tumor nuclei percentage, predicted DNA yield and size of tissue.

In conclusion, pathologist-directed NPE from tissue blocks elevated tumor purity, and consequently, increased proportion of successful reports and complex biomarker determinations from FFPE tumor blocks. By enhancing biomarker results, NPE may optimize patient matching to approved therapies and/or clinical trial enrollment while maximizing tissue preservation for additional tests. Moreover, this process is rapid, safe, inexpensive, and scalable. Precision punches may constitute best practice with respect to enriching tumor cells from low-purity specimens for biomarker detection in a routine molecular laboratory specimen-processing setting.

## Data availability statement

The original contributions presented in the study are included in the article/supplementary material. Further inquiries can be directed to the corresponding authors.

## Ethics statement

The studies involving humans were approved by Foundation Medicine, FMI Coded Research Protocol. The studies were conducted in accordance with the local legislation and institutional requirements. The ethics committee/institutional review board waived the requirement of written informed consent for participation from the participants or the participants’ legal guardians/next of kin because The data was de-identified with no PHI and research could not be practically conducted without waiving authorization.

## Author contributions

DL: Conceptualization, Data curation, Formal analysis, Investigation, Validation, Writing – original draft, Writing – review & editing. IL: Formal analysis, Investigation, Visualization, Writing – review & editing. RK: Data curation, Formal analysis, Visualization, Writing – review & editing. NP: Formal analysis, Investigation, Writing – review & editing. SL: Investigation, Writing – review & editing. BD: Formal analysis, Investigation, Writing – review & editing. TJ: Investigation, Writing – review & editing. DM: Investigation, Writing – review & editing. JR: Investigation, Writing – review & editing, Supervision. J-AV: Investigation, Supervision, Writing – review & editing, Conceptualization. JE: Investigation, Supervision, Writing – review & editing, Conceptualization. RH: Investigation, Writing – review & editing, Data curation, Writing – original draft. RSH: Investigation, Writing – review & editing. PM: Investigation, Writing – review & editing. JK: Conceptualization, Data curation, Formal analysis, Investigation, Methodology, Project administration, Supervision, Validation, Visualization, Writing – original draft, Writing – review & editing.
